# Necrotizing fasciitis: literature review of contemporary strategies for diagnosing and management with three case reports: torso, abdominal wall, upper and lower limbs

**DOI:** 10.1186/1749-7922-6-46

**Published:** 2011-12-23

**Authors:** Zdravko Roje, Željka Roje, Dario Matić, Davor Librenjak, Stjepan Dokuzović, Josip Varvodić

**Affiliations:** 1 Division of Plastic Surgery and Burns, University Hospital Centre Split, Croatia; 2 Department of Surgery, University Hospital Dubrava, Zagreb, Croatia; 3 Department of Surgery, University Hospital Centre Split, Croatia; 4 Department of Urology, University Hospital Centre Split, Croatia; 5 Department of Orthopedic Surgery, University Hospital Dubrava, Zagreb, Croatia; 6 Deparment of Neurosurgery, University Hospital Dubrava, Zagreb, Croatia

## Abstract

Necrotizing fasciitis (NF) is an uncommon soft tissue infection, usually caused by toxin-producing virulent bacteria. It is characterized by widespread fascial necrosis primarily caused by *Streptococcus hemolyticus*. Shortly after the onset of the disease, patients become colonized with their own aerobic and anaerobic microflora from the gastrointestinal and/or urogenital tracts. Early diagnosis with aggressive multidisciplinary treatment is mandatory. We describe three clinical cases with NF. The first is a 69 years old man with diabetes mellitus type II, who presented with NF on the posterior chest wall, shoulder and arm. He was admitted to the intensive care unit (ICU) with a clinical picture of severe sepsis. Outpatient treatment and early surgical debridement of the affected zones (inside 3 hours after admittance) and critical care therapy were performed. The second case is of a 63 years old paraplegic man with diabetes mellitus type I. Pressure sores and perineal abscesses progressed to Fournier's gangrene of the perineum and scrotum. He had NF of the anterior abdominal wall and the right thigh. Outpatient treatment and early surgical debridement of the affected zones (inside 6 hour after admittance) and critical care therapy were performed. The third patient was a 56 year old man who had NF of the anterior abdominal wall, flank and retroperitoneal space. He had an operation of the direct inguinal hernia, which was complicated with a bowel perforation and secondary peritonitis. After establishing the diagnosis of NF of the abdominal wall and retroperitoneal space (RS), he was transferred to the ICU. There he first received intensive care therapy, after which emergency surgical debridement of the abdominal wall, left colectomy, and extensive debridement of the RS were done (72 hours after operation of inquinal hernia). On average, 4 serial debridements were performed in each patient. The median of serial debridement in all three cases was four times. Other intensive care therapy with a combination of antibiotics and adjuvant hyperbaric oxygen therapy (HBOT) was applied during the treatment. After stabilization of soft tissue wounds and the formation of fresh granulation tissue, soft tissue defect were reconstructed using simple to complex reconstructive methods.

## Background

Necrotizing soft tissue infection (NSTI) is a rare but potentially fatal infection involving skin, subcutaneous tissue and muscle [1]. It is usually accompanied by the systemic inflammatory response syndrome (SIRS) and needs prolonged intensive care treatment [2]. Necrotizing fasciitis is characterized by widespread necrosis of the subcutaneous tissue and fasciae. However NF as a soft tissue infection *"per se" *typically does not cause myonecrosis, but does invade the deep fascia and muscle [3]. Its rapid and destructive clinical course is assumed to be caused by polymicrobial symbiosis and synergy [1,2]. Monomicrobial infection is usually associated with immunocompromised patients (cancer, diabetes mellitus, vascular insufficiencies, organ transplantation or alcohol abusers) [4]. Many aerobic and anaerobic pathogens may be involved, including *Bacteroides, Clostridium, Peptostreptococcus, Enterobacteriaciae, Proteus, Pseudomonas, and Klebsiella*, but group A hemolytic streptococcus and *Staphylococcus aureus*, alone or in synergism, are the initiating infecting bacteria [5]. Typical sites of the infection are the extremities, (primarily the lower extremities), abdomen, and perineum [1]. In most NSTI cases anaerobic bacteria are present, usually in combination with aerobic gram-negative organisms. They proliferate in an environment of local tissue hypoxia. Because of lower oxidation-reduction potential, they produce gases such as hydrogen, nitrogen, hydrogen sulfide and methane, which accumulate in soft tissue spaces because of reduced solubility in water [6]. Establishing the diagnosis of NF (as the most common type of NSTI) can be challenging. Clinical findings may include swelling, pain, fever, erythema, induration, crepitations, sloughing off of the skin, or a blistering and purulent collection. The need for more rapid and scientific methods of NF diagnosis led to the development of a clinical scoring systems, like the LRINEC scoring system (The Laboratory Risk Indicator for Necrotizing Fasciitis) or the APACHE II scoring system (The Acute Physiology and Chronic Health Evaluation) [6,7]. Unfortunately, still the hallmark NF symptoms are intense pain and tenderness over the involved skin and underlying muscle [6]. Because NF is a surgical emergency and a life-threatening condition, the patient must be admitted to an ICU, where start IC therapy and where immediate and aggressive surgical debridement must be performed [8]. The purpose of this review is to present the basic concept of the diagnosis and treatment of NF during the last few decades, as well as the contemporary strategies for diagnosing and management.

## Cases reports

### Case I

A 69 years old, diabetic (type II) male was admitted to the Emergency department (ED) because of a four day history of fever, vomiting and nausea (Table 1). We found abscesses on the posterior chest wall (CW), the right shoulder and arm. His diabetes mellitus was treated with oral anti-diabetic drugs. He had swelling and erythema of the affected skin and was warm to palpations. In the central zone there was sloughed off skin with a big circle of necrosis and crepitations. He had strong pain in the abdomen which appeared bloated, with strong peristaltic action and diarrhea. Oliguria with dark urine was also present. His laboratory blood values showed a non-regulated diabetes mellitus with hyperglycemia of 32 mmol/L, white blood cell count of 18 × 10^9^/L with 81.6% polymorphonuclear cells (PMNs), elevated C-reactive protein (CRP), hemoglobin, sodium and creatinine. His clinical picture indicated a state of bacterial sepsis and systemic toxemia. Ultrasonography showed reactive lymph nodes in both axillary regions and fluid collections on the posterior CW and the right arm. Anteroposterior chest x-ray revealed lung a shadow suggestive of inflammation in the basal level on the right side.

**Table 1:** Clinical findings in three case reports

Clinical findings	First case: 69 yr/M DM-type II, with NF of CW, shoulder, and arm	Secound case: 63 yr/M DM-type I, paraplegic with Fournier's gangrene	Third case: 56 yr/M with inquinal hernia repair and NF of AW and RP space
Preexisting medical conditions	DM type-II, hypertension, alcohol abuse, heart disease, peripheral vascular and pulmonary disease, malnutrition, chronic wound (pressure sores, diabetes and venous ulcer)	DM type I, hypertension, paraplegia, obesity, heart disease, peripheral. vascular and pulmonary disease, immune deficiency, pressure sores	hypertension, alcohol abuse, peripheral vascular disease

Physical findings	swelling, erythema, redness, induration, crepitus, pain, fever, warm skin, blisters, skin discoloration, numbness, soft tissue emphysema, confusion, weakness, skin sloughing/necrosis	induration, pain, crepitus, fever, warm skin, blisters, skin discoloration, soft tissue emphysema, paraplegia confusion, numbness	swelling, erythema, redness, induration, crepitus, pain, fever, warm skin, blisters, soft tissue emphysema, confusion, weakness, skin sloughing/necrosis

Vital sings and laboratory valves	SIRS and signs of systemic toxicity, positive LRINEC scour system.	SIRS and signs of systemic toxicity, positive LRINEC scour system.	SIRS and signs of systemic toxicity, positive LRINEC scour system

Source of infection	skin abscess/furunculosis	perineal abscesses, Fournier's gangrene	inguinal hernia repair, bowel perforation.

Microbiology findings	aerobes and anaerobes	aerobes and anaerobes	aerobes and anaerobes

Treatment modalities:			
primary debridement	yes	yes	yes
operative intervals (days):			
admission to first debridment	3 hours	6 hours	72 hours
first to last debridment	2	5	5
first debridement. to final closure	14 days	12 days	12 days
days to granulation tissue formation	7 days	10 days	10 days
hydrofiber dressing	yes	yes	yes

Adjuvant HBO therapy	yes	yes	yes
HBO sessions	4 sessions	11 sessions	11 sessions

Combination of antibiotics used	Penincillin G, Clindamycin, Imipenem, Teicoplanin	Penicilin G, Gentamycin, Clyndamicin	Penicilin G, Gentamycin, Clyndamicin, Metronidazol

Outpatient treatment	oral anti-diabetic drugs, antihypertensive drugs, cardiotonics	Insulin therapy, antihypertensive drugs, cardiotonics, different types of peroral antibiotics for 2 months	antihypertensive drugs, cardiotonics,

ICU therapy	dominantly mechanical ventilation, nutritional support, whole blood, fresh frozen plasma, erythrocyte concentrate, combination of 4 antibiotics (AB) which depending on wound culture or blood culture (administered for 10 days and target AB for 18 days)	dominantly dialysis, nutritional support, blood whole blood, fresh frozen plasma, erythrocyte concentrate combination of 3 antibiotics which depending on wound culture or blood culture (administered for 10 days and target AB for 11 days)	dominantly nutritional support whole blood, fresh frozen plasma, erythrocyte concentrate combination of 4 antibiotics which depending on wound culture or blood culture (administered for 14 days)

Main complications	delay in diagnosis and first debridement, inadequate serial debridement's, bacteriemia, sepsis, wound infection (MRSA), pressure sores, skin graft lysis	delay in diagnosis and first debridement, inadequate serial debridement, bacteriemia, sepsis, MODS, wound infection-MRSA, skin graft lysis, diverting colostomy, pressure sores	delay in diagnosis and first debridement, inadequate serial debridement, bowel perforation, bacteriemia, sepsis, secondary peritonitis, MODS, wound infection(MRSA), diverting colostomy, pressure sores

Reconstruction	skin grafts (SG), local flaps, topical negative pressure therapy with SG	skin grafts, local flaps, topical negative pressure therapy with SG, component separation technique with biological mesh	direct sutures, local flaps, component separation technique with biological mesh

Because of progress of systemic signs of soft tissue bacterial infections with septicemia and SIRS, early fluid resuscitation was started in the Emergency department. The metabolic changes, such as hyperglycemia and keto-acidosis, were also treated, and intravenous antimicrobial therapy (Penicilin G, Clindamycin, Imipenem, Teicoplanin) was begun. Surgical treatment was performed shortly after admittance in ICU. We applied an immediate and aggressive surgical debridement of the posterior CW, right shoulder, and right arm, with extensive fasciotomy on the arm. All infected and necrotic skin and subcutaneous tissue were radically excised up to bleeding healthy edges. Tissue cultures and fluid aspirate were sent for immediate gram-stain and culture. All opened wounds were copiously irrigated with hydrogen peroxide, saline and an antibiotic dressing of 1% povidone iodine solution was used to cover the wound. Next, exploration was performed after 24 hours, and all ongoing infected tissue was excised. Wounds were monitored during the next 72 hours with twice daily dressing changes. During the next five days, adjuvant HBO therapy in a hyperbaric chamber was applied. On the first day, the patient received two treatments of HBO therapy, and subsequently one treatment daily during the next four days. HBO was given at 2.8 atmospheres absolute pressure (ATA) for 90 minutes per day. We performed two additional debridements and one necrectomy for wound stabilization. After four days, microbiological analysis indicated a necrotizing infection with mixed aerobes and anaerobes. The dominant flora was *Peptostreptococus spp, Bacteroides spp and Fusobacterium spp*, though *Streptococcus pyogenes *and *Staphylococcus aureus *were also found. Blood culture was positive for methicillin-resistant *Staphylococcus aureus (MRSA)*. The wound stabilized and fresh granulation tissue appeared after seven days, at which point a second defect reconstruction was performed using skin flaps, skin grafts, and topical negative pressure therapy with skin grafts. The patient made an encouraging recovery from a NF affecting such a large area of the body. We believe that this was possible because of the multidisciplinary team approach involving a general practitioner, general and plastic surgeons, radiologist, microbiologist, physiotherapist and nutritionist. The patient was discharged after 32 days of hospital stay. Five months later he had regulated diabetes, and sufficient CW movement with good respiration rate, and normal range of motion in the shoulder joint and arm.

### Case II

A 63 years old, paraplegic and diabetic (type I) male patient was admitted to the Emergency department because of a two week history of high fever, perirectal pain, purulent drainage and a clinical picture of bacterial sepsis (Table 1). His diabetes mellitus was treated with insulin injections. He had pressure sores on the greater trochanter of right leg and sacral region which were treated with serial debridements and drainages on an outpatient basis by his family doctor during the previous two months. In his acute clinical status we found perianal induration with perianal abscesses and large grade III/IV sacral and trochanteric pressure sores, with multiple drainage sinuses. In both inguinal regions the patient had erythema and crepitations, stronger on the left side. The scrotal skin region was painful, edematous, and pruritic. On the left knee region there was an additional pressure sore with edema, fluid collections and lymphangitis in the ipsilateral inguinal region. His laboratory blood values showed signs and symptoms of SIRS with hyperglycemia of 21 mmol/L, a total leukocyte count of 6.35 × 10^9 ^with 78% of PMNs, 17% bands, 11% lymphocytes, and 120 × 10^9^/L platelets, hemoglobin of 78 g/L, albumin 29 g/L, protein 61 g/L CRP of 69,1 mg/L, and creatinin 216 mmol/L. The patient was febrile, with symptoms of systemic toxicity. In his local status he had scrotal gangrene, fulminating perineal abscesses and a fluid collection with crepitations on the left thigh. The plain film radiography of the pelvic region showed the presence of gas in the perineum. CT scan of the left thigh revealed suspected septic arthritis secondary to the pressure sore in the knee region, and low attenuation in vastus lateralis muscle, and gas in both perineal regions. The diagnosis of Fournier's gangrene was reached based on clinical examination and laboratory findings.

After admittance to the Emergency department, we started treatment with aggressive fluid resuscitation, correction of laboratory parameters, hyperglycemia, metabolic acidosis, adding an empirical combination of antibiotics-Penicillin G, Gentamycin, and Clindamycin. The first debridement was performed on the perineum area and continued to the scrotum, inguinal regions, and the lower abdominal wall (AW). We also performed an endoscopic lavage of the knee joint and fasciotomy, with radical debridement, of the thigh anterior compartment of the left thigh. The anterior compartment was opened from inguinal ligament to just above the knee joint. All opened wounds were copiously irrigated with hydrogen peroxide, 0,9% physiologic solution and dressed with 1% povidone iodine solution. After the initial debridement, the wounds were carefully monitored during the next 24 to 72 hours and dressing changes were done twice daily. Adjuvant HBO therapy was applied over the course of the next seven days. On the first day, the patient received two treatments of HBO therapy, followed afterwards by one treatment daily. HBO was given at 2.8 ATA for 90 minutes per day. We performed three additional debridement and necrectomy procedures to stabilize the wound. The fecal incontinence was treated with a diverting colostomy. The results of microbiological analysis of the perineum and thigh cultures showed a polymicrobial infection with *Escerichia coli*, *Psudomonas aeruginosa*, and *Streptococcus pyogenes*, and the presence of mixed anaerobes, including *Bacteroides fragilis*. Blood cultures were positive for *Pseudomonas aeruginosa*. Debridement and necrectomy was done with large skin defect on the left thigh and the lower AW. In the course of next ten days, the wound stabilized and fresh granulation tissue formed. At this point, a second defect reconstruction was performed using local flaps, skin grafts, topical negative pressure therapy with skin grafts and the technique of component separation with a biological mesh for ventral hernia repair. The temporary diverting colostomy helped in the healing of skin grafts which were used to cover soft tissue defects. The paraplegia was an additional daily problem for the patient's hygiene. Pressure sores on the sacral and trochanter region were treated longer than other skin defects. Six months later the patient had regulated diabetes. All defects were closed secondarily except for the sacral pressure sore which was treated as a chronic wound.

### Case III

A 56 years old healthy male patient was admitted to the Urology department for elective right inguinal hernia reparation (Table 1). The urologists performed a standard operation of a sliding inguinal hernia on the right side. Due to the weakness of the lower AW, the urologist reinforced the inguinal wall with synthetic Prolene mesh. Postoperatively, the patient showed a clinical picture of an acute abdomen. At this point, the urologists performed a revision surgery of the operated inguinal hernia, during which they found only a hematoma, removed the Prolen mesh and performed adequate haemostasis. Unfortunately they did not notice the bowel perforation and did not perform an explorative laparotomy at that time. During the next 24 hours, signs of septic shock with crepitations on the AW and right flank region appeared in the clinical picture. Through the suture line of the inguinal canal a fecal collection was drained. Postoperatively, the patient received a combination of Penicillin G, Clindamycin, Metronidazol and Gentamycin. The native abdomen x-ray showed air under the diaphragm. Magnetic resonance images provided dramatic evidence of an inflammatory process infiltrating the deep fascial plane of the anterior AW. Systemic manifestations of SIRS with body temperature more than 39°C, heart rate more than 100 beats per minute, breaths less than 30 per minute, PaCO_2 _less than 32 mmHg and WBC account more than 18 × 10^9^/L with a high number of immature forms, hypotension, hypoperfusion with a high level lactic acidosis, oliguria, and alteration of mental status and consciousness were indicators of severe sepsis and septic shock. The anesthesiologist introduced a central venous catheter and started intensive resuscitation.

The abdominal rigidity suggested a persisting peritonitis and an urgent laparotomy was done. Through a long midline incision we found a perforation of the caecum, necrosis of a great part of ascending colon, diffuse fecal peritonitis and signs of retroperitoneal NF. The surgical team executed extensive debridement, fasciectomy of the deep fascia on the AW, right orciectomy, right hemicolectomy, diverting colostomy on the descending colon and extensive debridement of the RS. The abdominal cavity and RS were extensively irrigated with hydrogen peroxide, saline and a solution of 1% povidone iodine, and drained on both sides. The structural and functional continuity of musculofascial system of the AW was obtained by component separation techniques (cite) and biological mesh. The wound was dressed with 1% povidone iodine solution. Dressing was controlled every 24 hours and serial debridements were performed. On the second postoperative day, the surgical team ordered adjuvant HBO therapy during the next 10 days. On the first day, the patient received two treatments of HBO therapy, followed by one treatment per day. HBO was given at 2.8 ATA for 90 minutes per day. In this case we needed five serial debridements to stabilize the wound. The results of microbiological analysis of the lower AW and retroperitoneal space showed a polymicrobial infection with *Escerichia coli*, *Psudomonas aeruginosa*, and *Streptococcus fecalis*, *Streptococcus pyogenes*, and the presence of mixed anaerobes, including *Bacteroides fragilis and Clostridum spp*. Blood cultures were positive for *Escerichia coli *and *Pseudomonas *aeruginosa. Methicillin-resistant *Staphylococcus aureus (MRSA*) was present in the second blood culture. Two weeks after the initial operation, the AW became stable and fresh granulation tissue appeared. At that point, we started closing the defects by using local advancement flaps, regenerative tissue matrix, and skin grafts. The closure of the diverting colostomy was performed three months postoperatively when the anterior abdominal has been strongly reinforced with a dermal matrix that was incorporated under the skin flaps. During long term follow up the colostomy was completely closed and regular bowel function was restored.

### Incidence and classification

Necrotizing fasciitis, the most complicated and life threatening NSTI, has a progressive and rapidly advancing clinical course [1]. Although occurring in all age groups, NF is slightly more common in older age groups (> 50 years of age) [2]. The infection usually affects the deep fascial plane, with secondary necrosis of subcutaneous tissue and skin caused by the thrombosis of the subcutaneous and perforators vessels. The incidence of NF has been reported to be 0.40 cases per 100 000 adults [3]. There is a male to female ratio of 3:1 in all cases of NSTI, which relates predominately to the incidence of Fournier's gangrene of the perineum [3]. The terminology used for infections of skin and skin structures is often confusing. Skin and soft tissue infections (SSTIs) are best classified according to the anatomical site of infection, depth of infection, microbial source of infection, or by severity (minor superficial lesion to invasive, fulminant and even lethal infections) (Table 2.). The Infection Disease Society of America made practical classification of SSTIs into three groups: superficial, uncomplicated infection (includes impetigo, erysipelas and cellulitis), necrotizing infection; infections associated with bites and animal contact; surgical site infections and infections in the immunocompromised host [3]. The recent clinical classification distinguished four NF types: Type I (70-80%, polymicrobial/synergistic), type II (20% of cases; usually monomicrobial), type III (gram-negative monomicrobial, including marine-related organisms) and type IV (fungal) [1]. Although NF is rare, its mortality rate is high, ranging from 6% to 76%, although it is much lower in recent studies (approximately 26%) [5]. Establishing the diagnosis can be challenging. Every physician must know the answers to four main questions: "What is the clinical course of NSTIs, especially of NF?", "Which types of organisms are responsible for the infection?", "What is the depth of the infection?", and "Is NF a life or limb threatening disease?". The first answer ensures early diagnosis of NSTI/NF, the second determines the empirical spectrum of antimicrobial therapy, and the last two answers point out the timing and the extent of surgical intervention.

**Table 2:** Classification scheme of skin and soft tissue infections (SSTIs) according to Sarani et al.[5]

Classification characteristic	Most common disease (underline) Incidence (%)
**Anatomic localization**	Fournier's gangrene of perineum and scrotum

**Depth of infection**	Necrotizing adiposities
	fasciitis, myonecrosis

**Microbial cause**	Type I: polymicrobial/synergistic/70-80% of cases
	Type II: monomicrobial (*Staphylococcus, Streptococcus, Clostridia spp*)/20% of cases
	Type III: marine related organisms
	Type IV: fungal

**Severity of infection**	
Uncomplicated infections	Superficial: impetigo, ecthyma
	Deeper: erysipelas, cellulitis
	Hair follicle associated: folliculitis, furunculosis
	Abscess: carbuncle, other cutaneous abscesses
Complicated infections	Secondary skin infections
	Acute wound infection (traumatic, bite related, postoperative)
	Chronic wound infections (diabetic wound infection, venous stasis ulcers, pressure sores)
	Perineal cellulitis with/without abscess

**Necrotizing fasciitis**	
Polymicrobial fasciitis (Type I)	Fournier's gangrene, synergistic necrotizing cellulitis with fasciitis and myositis
	Streptococcal gangrene
Monomicrobial fasciitis (Type II)	Marine-related organisms-*Vibrio vulneriformis *and other *Vibrio spp*
	Fungal spp

**Myonecrosis**	
Crepitant myonecrosis	Clostridial myonecrosis (traumatic gas gangrene and atraumatic gas gangrene-*Clostridium perfrigens and other Clostridial spp*)
	Synergistic necrotizing cellulitis with fasciitis and myositis
Non-crepitant myonecrosis	Streptococcal gangrene with myonecrosis-*Aeromonas hydrophila *myonecrosis

The causes of NF on the extremities are usually related to trauma, chronic wound infections, diabetes and vascular insufficiency, venous, diabetic and pressure sores, obesity, alcoholism, smoking, chronic liver disease, immune-suppression, or extravasation of drugs. This condition very often has a fatal outcome and many cases require amputation of an extremity rather than excision of the affected tissue to prevent proximal spread [6-9]. Delay in treatment of more than 6 to 12 hours or inadequate primary surgical debridement contribute to morbidity and mortality. The infection usually spreads rapidly along the fascial planes, accompanied by the production of particularly destructive bacterial enzymes that cause necrosis and liquefaction of the surrounding tissues. Crepitations and gas bubbles in soft tissue may be present. Published mortality reports with this conditions range between 8.7% and 73% (mean 32.2%) [3]. Unfavorable prognostic factors include old age, peripheral vascular insufficiency, and diabetes (Table 3.). Patients with diabetes appear to be particularly at great risk, representing over 70% of cases in one large review [10].

**Table 3:** Risk factors for development of NSTI and the LRINEC scoring system for prediction of NSTI

Risk factors		LRINEC scoring system	
	**Variable**	**Values**	**Score**

**Preexisting conditions**	C-reactive protein	≤150 mg/L	0
diabetes, immunosupression		> 150 mg/L	4
alcoholism, peripheral vascular disease, IV drug abuse, hypertension, corticosteroids, HIV, age < 50 years, GI malignance, malnutrition, major trauma, surgery, perforated viscera, chronic live disease, chronic renal insufficiency, obesity	White blood cell count	< 15 per mm^2^	0
		15-25 per mm^2^	1
		> 25 per mm^2^	2
	Hemoglobin	≤13,5 g/dL	0
		11-13,5 g/dL	1
		< 11 g/dL	2
	Sodium	≥ 135 mmol/L	0
		> 135 mmol/L	2
**Existing illness and injuries**	Creatinine	< 141 μmol/L	0
*Varicella *with bacterial superinfection, fractures, liposuction, seawater-seafood, surgery, spider bite and other bites, Cesarean section, burns		> 141 μmol/≤L	2
	Glucose	≤10 mmol/L	0
		> 10 mmol/L	1

NSTI-necrotizing soft tissue infection; GI-gastrointestinal; HIV-human immunodeficiency virus;

LRINEC-Laboratory Risk Indicator for Necrotizing Fasciitis: A score of ≥ 6 is suspicious for NSTI, a score of ≥8 is highly predictive of NSTI

The causes of NF on the CW are usually related to some form of trauma, tumor resection, irradiation or surgical procedure. The incidence of sternal wound infection with osteomyelitis after median sternotomy is 0.4% to 5.9%, and mortality is as high as 70% in infected patients [11]. Tube thoracostomy for empyema is a particularly noteworthy cause where the mortality is about 89%, which is approximately twice as high t as that reported for other anatomic sites [4,12]. Delay or inadequate surgical debridement and severity of the underlying thoracic condition, are responsible for the high mortality rates. The importance of early, aggressive and often serial surgical debridements with removal of one or more ribs cannot be overemphasized [11].

Fournier's gangrene in elderly patients and diabetics is usually described as a fulminating infection of the inguinal region and the lower AW and the perineum along with the scrotum and penis in men, and the vulva in women. Fournier originally reported a disease that was idiopathic in nature, but many recent studies suggest a polymicrobial etiology of this disease. The idiopathic causes are seen very often in younger populations [13]. The main sources of infection are elective skin operations, skin abscesses and pressure sores. The frequent colorectal disease includes anorectal infections, ischiorectal abcesses, colon perforations, and some elective anorectal diagnostic procedures e.g., rectal biopsy, anal dilatation, or hemorrhoidal banding. A genitourologic cause of the disease includes urethral stricture, trauma from an indwelling Foley catheter and prostate biopsy. In women, Bartholin abscesses and vulval skin infections are the most common causes of NF. Surgical management includes wide incision and debridement of all involved areas. As the involvement of deep fascia and muscles is rare with this syndrome, it is not necessary to continue the debridement into the healthy-looking tissue. The mortality ranges from 11% to 45% despite the improvement in critical care, usage of broad-spectrum antibiotics and aggressive surgical debridement [13].

The types of necrotizing infections on the AW are numerous and the indication for AW reconstruction after serial surgical debridements and necrectomies depends on the etiology, size and site of the defects. Complicated intra-abdominal infections such as appendicitis with perforation, infections after complex hernia repairing, perforated diverticulitis, cholecystitis, gastroduodenal perforations, small bowel perforations, obstructive colon cancer with perforation and complex trauma of the AW, are the main sources of NF in the AW and RS. Severe sepsis and septic shock can lead to multiple organ dysfunction syndromes (MODS). The defects of any size on the anterior AW may allow herniation of the viscera, which can lead into incarceration, and ultimately, strangulation. Any surgical incision can potentially result in ventral hernia, especially if a history of infection in that area is already present. Intra-abdominal infections "per se" include many pathological conditions, ranging from uncomplicated appendicitis to complicated fecal peritonitis [14,15]. Generally speaking, the choice of the surgical procedure depends on the anatomical source of infection, the degree of peritoneal and retroperitoneal inflammation, generalized septic response and patient's general conditions.

Retroperitoneal phlegmon with necrotizing fasciitis is an uncommon soft tissue infection that may become fatal. It usually ensues in cases of immunocompromised patients or advanced neoplastic disease. The infection spreads quickly and any delay in surgical intervention is associated with increased mortality. Necrotizing fasciitis of the anterior AW or perineum usually manifests with erythema and induration of the overlying skin. Nevertheless, when the retroperitoneum is involved, excision may be delayed due to the lack of clinical symptoms. Although the mortality rate of this infection is very high, survival is possible owing to the prompt and repeated wide surgical debridements and extensive necrectomy with proper broad spectrum antibiotic therapy [15,16].

### Risk factors

The most common risk factor for the development of NSTI is diabetes mellitus, with an occurrence of 56% in all cases [7,17] (Table 3). The other co-morbidities include obesity, alcohol abuse, immunodeficiency, chronic renal failure, liver cirrhosis, hypertension, peripheral vascular disease, and age above 60 years. When such conditions occur simultaneously, the mortality rate increases remarkably on average 64,7%. [17,18]. According to this, the incidence of these infections is rising because of an increase in the number of immunocompromised patients, diabetes, cancer, alcoholism, vascular insufficiencies and organ transplants. Almost half of these infections are idiopathic, because we are not able to identify any underlying lesion at the site of the NSTI [7]. The best examples of such cases are scrotal or penile NF. Causative organisms are numerous and often may be polymicrobial (Table 3) [18,19].

There is no age or sex predilection for infection [18]. Because of the accompanying systemic illness and profound tissue inflammation, these patients are usually critically ill and have prolonged ICU stay. They need critical care therapy and complex surgical management, and can be treated in a specialized facility such as a burn center or a burn unit [7]. Laboratory based scoring systems as LRINEC score test (The Laboratory Risk Indicator for Necrotizing Fasciitis) [20] (Table 3.) or APACHE II score test (The Acute Physiology and Chronic Health Evaluation) may help in the early diagnosis of NF [21]. Both scoring tests are not NSTI specific, but are accurate predictors of mortality rates in most NF cases.

### Pathophysiology and microbiological findings

According to the updated consensus for NSTIs (1,2), microbial invasion of skin and subcutaneous tissue occurs either through external trauma and surgical wounds, or directly through bacterial invasion from a perforated viscus. Table 4 present potential antibiotic therapeutic regimens for certain pathogenic organisms and predisposing factors. Microorganisms appearing in the skin and subcutaneous tissue spaces produce various endo- and exotoxins that cause prolonged vasoconstriction in the dermal capillary network. When these toxins are released into the systemic circulation, they produce the SIRS, which can progress into septic shock, MODS and finally, death [1,2,14]. The central pathohistological point in the pathogenesis of NSTIs is the thrombosis of perforating vessels of the skin and subcutis [17]. As the spread and extent of infection do not correspond with overlying skin changes, an inexperienced surgeon might not clearly determine the seriousness and extent of infection that takes place under the skin surfaces and in the subcutaneous space. In case of fulminating NF, MODS will develop within the first 24 hours of infection. In this case the disease will very often become fatal if not promptly recognized and treated with extensive surgical debridement, appropriate a combination of the antibiotics, and intensive care resuscitation [21].

**Table 4:** Suggested potential antibiotics therapeutic regimens depending on pathogens organisms, clinical conditions, predisposing factors, and antimicrobial choices

Pathogens and clinical condition	Predisposing factors	Antimicrobial choices
**Gram-positive organisms**		
Group A streptococcus (*S. pyogenes*) Erysipelas	Minor skin trauma or skin break	penicillins or cephalosporins, or alternative therapy: clindamycin, macrolides, glycopeptidescephalosporins, semi-synthetic resistant penicillin or
Cellulitis	Minor skin trauma or break	alternative therapy: clindamycin, macrolides, glycopeptides
Necrotizing fasciitis with/without myonecrosis	Minor skin trauma or skin break, superinfection of varicella lesion, DM, non-steroid anti-inflammatory drugs	high dose penicillin G, clindamycin or alternative therapy: clindamycin
Group β streptococcus (*S. agalactiae*) Necrotizing fasciitis	DM, premature neonates	high dose penicillin G, clindamycin or alternative therapy: clindamycin
Community-acquired meticillin resistant; *Staphilococcus aureus (CO-MRSA)*	No specific risk factors	glycopeptides or clindamycin, or alternative therapy: linezolidin, sulfomethoxazole, clindamycin
	Nasocomial MRSA in health care facilities is the major risk factor	high dose penicillin G, clindamycin or alternative therapy: clindamycin, metronidazole
*Clostridium spp*	Gross tidy and contaminated wounds	
	(*C. perfrigens*)	
	Colonic contamination (*C. septicum*)	
	IV drug use (C sordellil, C nayvi)	

**Gram-negative organisms**		
*Pasteurella spp*	Dog bites (P canis) Cat bites (P multocida)	amoxicillin, clavulanate piperacillin, tazobactam, III-generation cephalosporin metronidasole or alternative therapy: clindamycin, flouroquinolone, trimoxasole
*Aeromonas spp (A. hydrophilia)*	Freshwater exposure, medical leeches	fluoroquinolones or alternative therapy: trimoxasole, cephalsporins, aminolgycosides
*Vibrio spp (V. vulnificus)*	Chronic liver disease, DM	minocycline, cephalosporine or alternative therapy: ciprofloxacin
*Klebsiella pneumonia*	Chronic liver disease, DM	cephalosporines, amoxicillin, carbapenems, flouroquinolones, or alternative therapy: amynoglycosides
*Escherichia coli*	Cirrhosis	cephalosporines, amoxicillin, carbapenems, flouroquinolones, or alternative therapy: amynoglycosides
*Serratia marcescens*	Chronic renal failure, DM	cephalosporines, amoxicillin, piperacillin, tazobactam, carbapenems, flouroquinolones, or alternative therapy: amynoglycosides
*Pseudomonas aeruginosa*	Neutropenia, haematological malignancy, burns, HIV infection, injection drug use	amoxicilin, aminoglycosides, or alternative therapy: flouroquinolones

From the clinical point of view, NF is usually a polymicrobial (Type-I) rather than a monomicrobial infection (Type-II) [18]. The analysis of our cases, during the *follow up *period of 15 years (36), point out that the most common bacterial species involved are: group A beta-hemolytic *Streptococcus pyogenes*, anaerobes (*Bacteroides, Clostridium, Peptostreptococcus*), group B *Streptococcus, Pneumococcus *and other *Streptococcus *species, *Staphylococcus aureus*, including hospital acquired *MRSA *and gram-negative enterobacteriaceae (*Escherichia coli, Acinetobacter species, Psudomonas, Serratia, and Klebsiella pneumniae*). In the retrospective study by Elliot et al. [17] they reviewed 198 patients with documented NSTI. These 182 patients grew an average of 4.4 types of microbes from original wound cultures, although a single pathogen was responsible in 28 patients. Eighty five patients had combined aerobic and anaerobic growth, the most common organisms being, *Bacteroides species*, aerobic streptococci, staphylococci, enterococci, *Escherihia coli*, and other gram-negative rods. Clostridial growth was common but did not affect mortality unless associated with pure clostridial myonecrosis. Mortality was affected by the presence of bacteriemia, delayed or inadequate surgery, and degree of MODS on admission. Monomicrobial cases are usually caused by Group A *Streptococcus pyogenes *and *Staphylococcus aureus*. They occur in otherwise healthy, young, immunocompetent patients and are most usually located on the extremities. In the study by Anderson et al. [22] more that 71% of cases had a polymicrobial source of infection. A polymicrobial infection is often diagnosed in immunocompromised patients and usually occurs in the perineum and trunk area [23]. Toxic shock syndrome is the most often accompanying syndrome of Streptococcal sepsis [24].

### Clinical findings

The most representative clinical picture present with abscesses, infected traumatic and surgical wounds, intravenous drug abuse, pressure sores burns, perforated viscera (particular colon, rectum, and anus), recently performed liposuction, infected vascular prostheses and grafts, and invasive cancer [18,19]. Early clinical suspicion and surgery are the keys to improving survival, and patients with necrotizing infections need an integrated multidisciplinary approach to management. It is adjusting with the infecting organism(s), the site of infection, and the effects from any toxins produced, and incorporate various clinical and laboratory parameters In everyday clinical practice a universal clinical guideline that should be used in the diagnosis and treatment of all types of NSTI/NF does not exist (Table 2, 4, 5).

**Table 5:** Treatment options classified by type of infection and clinical picture

Type of NSTI	Depth of involvement	Usual pathogens	Predisposing factors	Time of incubation and rate of progression	The main clinical signs	Treatment options
Polymicrobial NF-type I	fascia and muscle	obligate and facultative anaerobes	different type of wounds	long (48-96 h) Hour to days	foul- smelling drainage	ICU stay critical care therapy surgery antibiotics ev. HBO

Monomicrobial NF-type II (Steptococcal gangrene)	skin, fascia and muscle	Streptococci -groups A, C, G, and B; (B is more common)	excoriation or cut wound	short (6-48 h) A few hour	distinct margins	ICU stay critical care therapy surgery antibiotics ev. HBO

Gas gangrene (Clostridial myonecrosis)	muscle	C. perfirngens (C. perfirngens more common) and C. novyi	tidy wounds	short (6-48 h) A few hour	extreme system toxicity	ICU stay critical care therapy surgery antibiotics HBO
		C. septicum	gastrointestinal lesion			

Non-Clostridial myonecrosis	muscle and fascia	obligate and facultative anaerobes or *A. hydrophila*	different type of wounds	variable (12-96 h) Hour to days	gas in soft tissue	ICU stay critical care therapy surgery antibiotics HBO

The clinical findings important for establishing the early NF diagnosis can be divided into two groups, early and advanced symptoms [25]. Primary or idiopathic NF usually occurs in the absence of a known causative factor or entry site for bacteria spreading. On the other side, secondary NF is the result of a known etiology and takes place through laceration of skin, cut, abrasion, contusion, burns, bite, subcutaneous injection or operative incision. The most common early signs are erythema, local warmth, skin induration and edema. In the disease's fulminant form, the patient is critically ill with signs and symptoms of severe septic shock and MODS, and extensive spreading of soft tissue necrosis. The clinical picture worsens very quickly, practically during only a few hours [25,26]. The acute form of the infection spreads for a few days and it first begins with severe pain before the cutaneous manifestations appear. The subacute NF form has an indolent clinical course, which progresses slowly over days or weeks [25].

Early clinical status during the first 24 hours usually includes minor trauma, skin infection like folliculitis or abscess, gangrene on the extremities, pressure sore(s), or a complicated surgical incision like hernia repair. The external signs on the skin may be erythema or induration. The patient usually feels pain on the site of the injury. There is a disproportion between the character of the injury and intensity of the pain. Pain out of proportion with the apparent lesion severity should suggest a possible NF diagnosis [1,2].

During the next 2-4 days, the pain becomes more intense. In the clinical status we find many symptoms of general toxicity like fever, dehydration, confusion, dizziness, diarrhea, nausea, vomiting, weakness and malaise. If the patient is not admitted to an ICU or the diagnosis is established late in its course, more serious clinical symptoms ensue. The limbs and the area of body where the patient felt pain begin to swell, and may show a purplish rash or blisters with "dish-wash" purulent or haemorrhagic fluid. Cutaneous changes may be minimal, or may progress to blisters and bullae, and then to circumscribed necrosis of skin. Also, emphysema and gas formations with crepitations in overlying skin may appear. The pain grows, but remains an disproportionate to the clinical picture [1,5,6].

In the late phase within 4-6 days, symptoms of septic shock or MODS usually appear. Those symptoms may include cardiac shock with tachycardia, hypotension and decreased cardiac minute output, an elevated white blood cell count, metabolic acidosis, coagulopathy, changes in mental status and weakness. The patient in the late stage of NF appears apathetic and indifferent. If the patient has an accompanying illness like diabetes, obesity, alcoholism, malignancy, malnutrition or immune deficiency, the general status is compromised with additional symptoms of these illnesses [2,15].

As early clinical findings, in the course of our clinical cases, we especially emphasize tenderness, swelling, erythema, and pain [2]. Those clinical symptoms and signs are similar to the course of superficial cellulitis, and it is very difficult to establish an early diagnose of NF at that moment. Nevertheless, a high suspicion must be present in all cases of rapidly progressive cellulitis, associated with severe progressive pain [6]. The hallmark symptoms of NF on the perineum, extremities and posterior CW include intense pain and tenderness over the involved skin and underlying muscle [5,6,27]. Over the next several hours and days, local pain can progresses to anesthesia because all cutaneous nerves are destroyed, which depends on the extent of tissue necrosis.

It is particularly difficult to establish the diagnosis of NSTI in outpatient facilities, because many of concomitant co-morbidities are able to cover the true clinical picture of necrotizing infections. Misdiagnosing NF is particularly common in children, and usually associated with recent varicella-zoster infection [5,28]. The surgical exploration of the suspected infection site, combined with microbiological and histopathological analysis of 1 cm^3 ^of soft tissue, is the gold standard for establishing an early NF diagnosis [5].

Necrotizing infection of the AW with concomitant secondary peritonitis always presents a very challenging issue, especially when it appears after an unrecognized bowel perforation during inguinal hernia repair. The mortality rate associated with acute pancreatitis and concomitant retroperitoneal NF [5,29], metastatic gas gangrene with colonic perforation [5,30], intra-abdominal infection with severe sepsis or septic shock is approximately 30% [31]. The main prognostic factors for these patients include advanced age, poor nutrition, concomitant diseases, i.e. diabetes, vascular and chronic renal insufficiency, advanced septic shock, multiple organ failure, immunosuppressed host and nosocomial infection [6,32]. The clinical picture is characterized by intense abdominal pain, a brown discoloration and bullae of the abdominal skin, gases in the soft tissue, abdominal rigidity, additional RS NF and myonecrosis of the AW in cases of clostridium infection [5,6,33]. Indeed, early detection and radical surgical treatment is essential to minimize the morbidity rate and to save life [5,6,23].

The causative triggers for the development of Fournier's gangrene are urogenital, anorectal and cutaneous disorders [1,6,34]. Fournier's gangrene usually begins with pain and itching of the perineum and scrotal skin. In cases originating in the genital area, the infecting bacteria probably pass through Buck's fascia of the penis and spread along the Darto's fascia of the scrotum and penis, Colle's fascia of the perineum, and Scarpa's fascia of the lower AW. The additional necroses of the superficial fascia and fat produces a thin watery malodorous fluid and crepitance (usually associated with polymicrobial infections including *Enterobacteriaceae *and *Clostridiae spp*) are results in more evident signs of necrotizing infection. Patients with SIRS can have high fever, anxiety, altered mental status, leukocitosis, shock and tachypnea. In that particular case, when severe soft tissue infections is already suspected, the usage of the LRNIC scoring system for prediction of NF are very useful for exact diagnosis [2,20]. By the time the progression of clinical signs becomes obvious, the appearance is usually that of a late NF phase, with visible bruising, bullae and cutaneous necrosis due to the extension of the necrotizing process from the deep fascia and horizontal spread [1]. The case history at that moment should suggest the causative microorganisms of infection. Nevertheless, the lack of cutaneous findings early in the course of the disease makes the diagnosis more challenging, and a high suspicion is essential for each clinical sign that appears on the skin and subcutaneous tissue.

The accumulation of gas formation in the soft tissue, which is seen in half of all NF cases, is another cardinal sign of NF diagnosis. It is clearly visible on plain x-ray pictures. More useful clinical findings are visible with ultrasound, CT scan and MRI. We prefer an additional skin puncture with large gauge needles to mobilize gas from subcutaneous spaces. If we do not find any gas bubbles, but the clinical picture presents other relevant clinical signs of NF, we must perform a radical surgical debridement as soon as possible, and prescribe broad-spectrum antibiotics that cover aerobic and anaerobic microbial species [15,24].

### Diagnostic imaging modalities

The most important clinical signs of NF are tissue necrosis, putrid discharge, bullae, severe pain, gas formations in soft tissue, rapid spreading through fascial planes and the lack of classical tissue inflammatory signs, i.e. "*dolor, color, rubor, tumor and functio laesa*". Today, CT and MRI are superior methods compared to sonography, scintigraphy and plain radiography, which also provide useful information about the nature and the extent of necrotizing infection [1,2,35]. Nevertheless, physical examination and a clear understanding of the clinical picture are the most important means in establishing an early diagnosis of any type of NSTI and NF [6,36].

### Treatment

Successful treatment of NSTI requires a multidisciplinary approach from the onset and coordination between general practitioners and surgeons for outpatient cases, and between the surgeons and other specialists in hospital facilities. The first and economically most important decision in treating necrotizing infections concerns the need for hospitalization. Most pyodermas and uncomplicated skin and soft tissue infections do not require hospitalization. Complicated necrotizing infections often require admission, especially if fascia or muscle involvement is suspected. If the process is rapidly progressing, signs of systemic toxemia develop, the diagnosis or prognosis is in doubt, exploratory surgery is contemplated or the patient cannot adequately comply with outpatient treatment. These days NSTI and NF still exists as a life threatening soft tissue disease, therefore patient must be promptly admitted into a hospital ICU [6,37] in which appropriate treatment including radical surgical debridement of the entire affected area should be performed. The fluid resuscitation must be ordered immediately upon arrival, to maintain hemodynamic stability and vital functions.

Today, the generally agreed upon algorithm for care is: 1-Resuscitate the patient in shock; 2-Begin with broad spectrum antibiotics which cover polymicrobial infection; 3-Take patient to the operating room for early comprehensive debridement of all dead tissue. Doubt as to the diagnosis can be settled using frozen section histologic analysis. Obtain gram stain and culture from the wound; 4-Further debridement's should be repeated every 24 to 48 hours until the infection is controlled; 5-Antibiotic therapy should be adjusted to adequately cover organisms obtained on initial culture; 6-HBO can be considered in the hemodynamically stable patient, if available (Table 5).

A combination of antibiotics is the key to successful adjuvant therapy, most of our patients having been treated with empirical antimicrobial therapy before we established the early diagnosis of necrotizing infection. In the majority of our cases the wound cultures were collected at the time of initial surgery. Unfortunately, antibiotic therapy alone has little value because tissue hypoxia and ischemia do not permit adequate delivery of antibiotics to the target tissue [6,36]. The polymicrobial infection identified by wound cultures was the dominant causes of NF in our study (Table 1, 4). For that purpose we used a combination of antibiotics that cover a broad spectrum of anaerobes (Clindamycin) and aerobes, gram-positive (Penicillin G or extended spectrum Penicillin, Imipenem and Teicoplanin) and gram-negative organisms (Aminogliycosides, Cephalosporins, or Carbapenems) [36,38]. Our therapeutic regimen usually consisted of Penicillin G, Clindamycin and Gentamicin [36]. In cases when we used Aminoglycosides, renal function with creatinin excretion was additionally monitored. Because of the increasing number of MRSA infections, Daptomycin or Linezolid should be considered as part of the therapeutic regime, until MRSA infection has been excluded. Vancomycin is also in use, but it does not have any effect on exotoxin production [1,2]. For the anaerobes coverage we have provided some other combination of antibiotics like Metronidazole and third generation Cephalosporins [8,25,39]. After gram staining and culture for rapid bacteriologic diagnosis or rapidly frozen biopsy examination have been established, we started with targeted antibiotics. The culture-negative rate in our study was probably not due to the use of empirical antibiotic treatment before the wound culture was available, but it is lower than in other studies [36,40,41]. Unfortunately, contemporary dilemmas about how long to use antibiotics also exist. We recommend continuing with the antibiotic therapy for 3 to 5 days after the systemic signs and symptoms and most local signs of soft tissue infection have resolved. Other authors suggested the same approach [22,25,36,38].

The emergency surgical debridement of all affected tissue is the primary treatment modality for NSTI and NF. It includes prompt and radical surgical debridement, necrectomy and fasciotomy in cases presenting with the compartment syndrome [8,37]. Surgical intervention can be life-saving and must be performed as early as possible. Surgical procedures should be repeated during the next 24 h, 48 h, or longer, depending on the clinical course of the necrotizing infection and vital functions. Numerous studies [5] have shown that the most important variable for the mortality rate is the timing and extent of the first debridement. In the study of Mock et al. [42] the relative risk of death was 7,5 times greater in cases with improper primary debridement, and in the study of Wong et al. [43] it was 9 times greater when primary surgery was delayed more than 24 hours. Incisions are performed parallel to Langer's lines to ensure better surgical wound healing and less scaring [6,36]. We start the incision over the point of maximal fluctuation and then extended in the direction of Langer's lines. The surgery also minimizes the overall tissue loss because it cuts the way the infection spreads in course of facial plan and eliminates the need for amputation of the infected limbs [44]. After the release of pus and fluid by performing incisions which are parallel with Langer's lines we can perform additional perpendicular incisions on the skin [6] to maintain the wound open, and to allow free drainage and to remove additional necrotic tissue. But, skin bridges and flaps generally should be avoided while performing incisions. Every patient who has NSTI and NF needs a regular inspection of the operated wounds during the next 24 hours and later. If there is any concern about the tissue viability, the surgeon must promptly perform a re-operation with additional radical debridement. We maintain that the main reason for the progression of the infection lies in the delay of the first operative debridement, inadequate primary debridement and necrectomy, hemodynamic instability and concomitant illness [36]. The flow of intravascular liquid into third tissue spaces in each presented case was large and therefore hemodynamic resuscitation, nutritional support and enteral feeding in ICU must be started as soon as possible. These measurements also prevented concomitant catabolism [1,2,14,15].

During surgical intervention, the following signs are of greatest importance for the NF diagnosis: grayish necrotic deep fascia, a lack of resistance of normally adherent muscular fascia to blunt finger dissection *("Finger test")*, lack of bleeding from the fascia and the presence of dish-water pus [6,36]. Based on our surgical practice we also recommend an early and very aggressive debridement of all involved tissue that can be easily elevated off of the fascia with gentle pressure or finger spreading. The surgical intervention in which we removed all infected tissue in a single operation, rapidly improved the clinical course of the infection. All deep fascia and muscle should be inspected for potential involvement with streptococcal myositis or clostridium infection. We believe that the mass and the extension of soft tissue that must be initially excised depend on the body region in which the infection appeared. Nevertheless, the extent of debridement should not be needlessly limited because the novel plastic surgical techniques can cover every wound defect size.

Special attention should be paid to the upper and lower extremities, AW with intra-abdominal infection such as secondary peritonitis, and on the CW with persisting sternum infection and mediastinitis [8,11,26]. The extent of debridement is very important on the extremities. In cases with compromised viability and compartment syndrome additional fasciotomies of all fascio-cutaneous spaces should be performed [36]. A suspected case of clostridial myonecrosis requires early surgical exploration and extensive debridement of all involved muscle structures [36]. A tourniquet should be used during the limb surgery to reduce blood loss and offer better examination [36]. The incision proceeds proximally from the infected area in a longitudinal manner, until healthy fascia adherent to the overlying subcutaneous tissue and underlying muscle is encountered. In that moment, the tourniquet should be deflated, the wound checked to confirm tissue viability, and then meticulous hemostatis should be performed [36]. Still, controversy exists regarding how much tissue should be initially excised because the skin may often appear normal. Andreasen et al. [22] investigated the normal-appearing tissue microscopically, and found that soft tissue had extensive vascular micro-thromboses as well as vasculitis. Their finding indicated that this tissue, which has a normal external appearance, has a high risk of full thickness necrosis [22]. Poor prognostic indicators for limb amputation very often include old age, peripheral vascular disease and diabetes [36,44,45]. Amputation must be obligatory considered if the extent of infection includes a large joint and most muscle groups or if the infection is rapidly spreading towards the torso [36,46].

Postoperative wound management consists of serial dressing changes, until the wound becomes free of recurrent or progressive skin and soft tissue necrosis. The wound is washed and dressed with occlusive, adsorptive bandages with antiseptic two or three times a day [36]. We recommend daily antibiotic dressings such as 1% povidone iodine solution or 1% Silver sulfadiazine cream (Dermazin). Articoat and hydrofiber dressing like Aquacel Ag is also a useful method for the control of the infection, during the procedures of secondary wound defect closure [36,47]. After the initial surgery, the wound must be carefully examined in general anesthesia every 24 h, to assess the tissue viability and necrotizing infection progress [36,44]. Serial debridement must be performed more times (median range in our study was four times) because the necrotizing infection is rarely eradicated after a single debridement [36].

**Figure 1:**
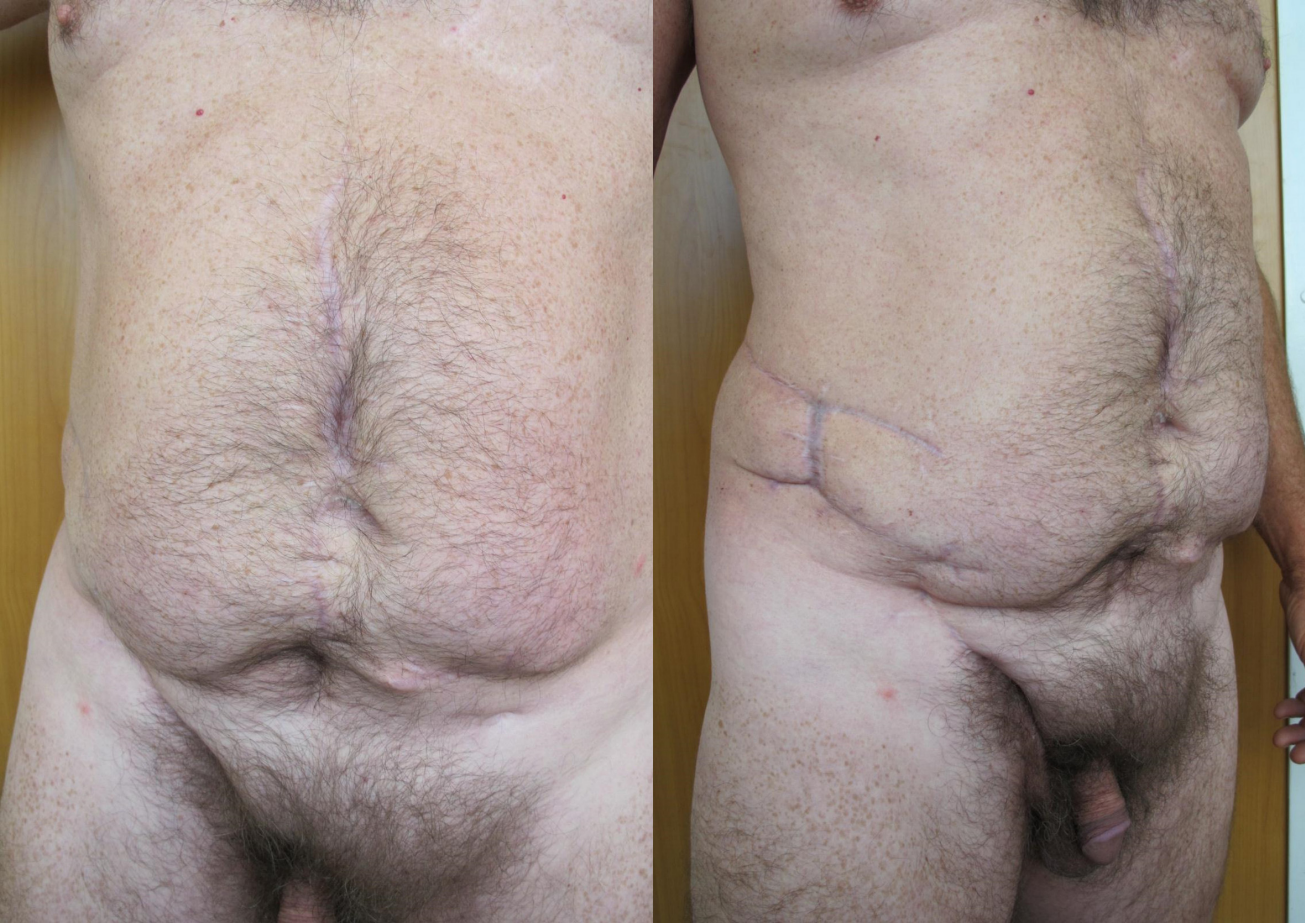
Postoperative view of Fournier's gangrene and necrotizing fasciitis of the abdominal wall with closed divergent colostomy.

Perineal, perianal, or scrotal infections require special consideration (Figure 1). In the presence of a pressure sore, perineal abscess or paraplegia, necrotizing infection spreads into the scrotum, inguinal region and lower AW. In some particular cases, it is necessary to perform a diverting colostomy, cystostomy, or both to facilitate the formation of granulation tissues and wound hygiene, and to protect the flaps or skin grafts healing process. Surgical management includes wide tissue incision, radical debridement with orchiectomy and drainage of all involved areas [13]. The wound is abundantly washed with hydrogen peroxide, saline and 1% povidone iodine solution. Finally, it is dressed with occlusive and adsorptive bandages with antiseptic, and changed twice daily. After the wound stabilizes and fresh granulations form, we perform secondary soft tissue defect reconstructions.

NF of the AW and RS, even today, presents a challenging surgical issue. Skin incision must be performed in the longitudinal direction along the muscle-fascial layers of the inner AW until healthy fascia adherent to the overlying subcutaneous tissue and muscle is encountered. It is not indicated to perform two, three or more parallel incisions or any perpendicular incisions, because the bridges of skin and skin islands will usually not survive. Postoperative wound management on the AW consists of serial dressing changes during the next 24 h to 48 h, until the wound is free of recurrent or ongoing infection. When infection progresses across the deep fascial plane of the AW or a necrotic area on the skin appears, aggressive surgical debridement should be repeated. In our case with NF of the AW and RS we usually performed two to five debridement procedures to stabilize the wound conditions. The primary defect on the AW is usually large and it is repaired with advancement flaps using an abdominoplasty technique, biological mesh or skin grafts [48]. These days, after the primary surgery of NF we can also use the topical negative pressure therapy (TNP) [49], artificial skin (Integra) [50], synthetic prosthetics [51] and bio-prosthetic (biologic) mesh [52,53]. In our particular case with caecum perforation during inguinal hernia repair, fecal peritonitis and septic shock were present. We performed explorative laparotomy via midline incision and found diffuse peritonitis, ischemia of small bowel and right colon, and NF of the RS. Published reports point out that ultrasound, native abdominal x-ray films or CT scanning are very useful preoperative diagnostic methods for bowel perforation with diffuse peritonitis, but the exact condition is always discovered intraoperatively [15,23]. We decided to apply a combination of antimicrobial therapy that covers aerobes and anaerobes. After we received the results of microbiological analysis, we ordered antibiotics for each causative organism. During the first operation we performed an extensive surgical debridement of the RS, right hemicolectomy, diverting colostomy on the left colon and multiple drainages of the infected intra-abdominal fluid collections. There is still controversy about the optimal surgical management of colonic perforation complicated with peritonitis. Hartmann's resection has been considered the procedure of choice in cases with diffuse peritonitis and remains a safe technique for colectomy in a perforated colon, especially in elderly patients with multiple co-morbidities [30,31]. More recently, some have suggested that primary resection with anastomosis is a modern approach, even in the presence of diffuse peritonitis [30]. After the wound is stabilized with fresh granulation tissue, we could perform a second reconstruction of the AW defects, primarily with advanced flaps and skin grafts. The diverting colostomy was closed in a third operation.

### HBO therapy

The use of HBO as an adjuvant therapy for NSTI is based on animal and human studies, and continues to be the subject of scientific analysis [45]. Several studies have shown decreased morbidity and mortality when HBO is used postoperatively as adjuvant therapy [26,36,45]. However, HBO should not interfere with or delay the repeated surgical debridement. The newest data indicate that oxygen administration in the perioperative period may reduce the risk of wound infection [36,54]. The reason for this is that the ability of neutrophil leucocytes to kill bacteria depends on the oxygen availability and formation of free oxygen radicals. HBO additionally increases oxygen diffusion into soft tissue and facilitates the synthesis of collagen and angiogenesis [54]. Better perfused tissue is more resistant to infection (especially from anaerobic spp.) [55] and exotoxin excretion by *Clostridium *spp. [56,57]. We have determined the effect of HBO therapy on short term complications of complex war wounds to the upper and lower extremities that included cases with NSTI and NF in patients who were and patients who were not treated according to the North Atlantic Treaty Organization (NATO) emergency war surgery recommendations [36]. We found that HBO therapy significantly reduced the time to wound stabilization and fresh granulation production, as well as the occurrence of wound complications (deep soft tissue infection, osteomyelitis, skin graft lyses and flap necrosis) in patients with Gustilo type III extremity war wounds. Also we have gained additional experience with the use of HBO therapy for severe life-threatening infections such as clostridial myonecrosis and other aerobic and anaerobic NSTI. Regardless of the type of surgical strategy applied, the HBO therapy should never delay the emergency of the surgical intervention, including the treatment of *Clostridium perfrigens *causing gas gangrene [36,54,57].

### Reconstructive surgery

The reconstruction of skin defects either on the extremities and torso, or on the abdominal or chest wall, should be performed using several different techniques and surgical materials on each patient. As is often seen, a complete loss of skin or dermal structures needs a complex, multilayer reconstruction especially in functional areas of the body and on the extremities. Novel concepts of layer-specific reconstruction include biologic meshes, which are an alternative to flap and skin graft surgery, especially in abdominal and chest wall reconstructions [58-61]. After the wound stabilizes and fresh granulation tissue without any signs of acute infection we perform staging reconstructions using simple to complex reconstructive methods. The main contributing factor for reconstructive method-selection was the extent and the localization of the defect and the patient's condition [51-53].

Topical negative pressure therapy has been reported to remove exudates, cover wounds securely, stimulate angiogenesis [6,49] and reduce bacterial contamination [50]. It also reduces the surface area of the wound, improves the rate of granulation tissue formation, reduces the number of surgical excision procedures needed, as well as enables better healing performance of skin grafts and biologic meshes. The cost benefit of that novel therapy is evident, but the complications of TNP still exist and include damage to surrounding tissue due to pressure effects, pain during dressing changes and discomfort because of very bulky dressing [52]. Newer data recommend the use of TNP in the acute traumatic military settings [58]. Leininger at all used TNP in the deployed military settings (at R3 stage of-NATO medical care) where they treated all Iraqi casualties with TNP dressing after their first debridement (77 cases) [59]. They reported that infection rates dropped from 81% to 0% after using the TNP management strategy. Our experience has shown the use of this wound management technique to remove exudates, improve the patient comfort, reduce the wound size and the time for wound stabilization, to allow the formation of fresh granulation tissue, and better healing of skin grafts and flaps [36].

Reconstructive options on the extremities start as simple and advance towards the more complex as needed for a given defect. The reconstructive ladder is a useful way to systematically plan the closure of any wound on the extremities [36]. The reconstructive ladder begins with healing by secondary intention as the base level, and advance with primary closure, skin grafting, local flaps, regional flaps and free tissue transfer. The final methods for extremity reconstruction are the use of TNP and perforator flaps (Table 1) [50-53].

**Figure 2:**
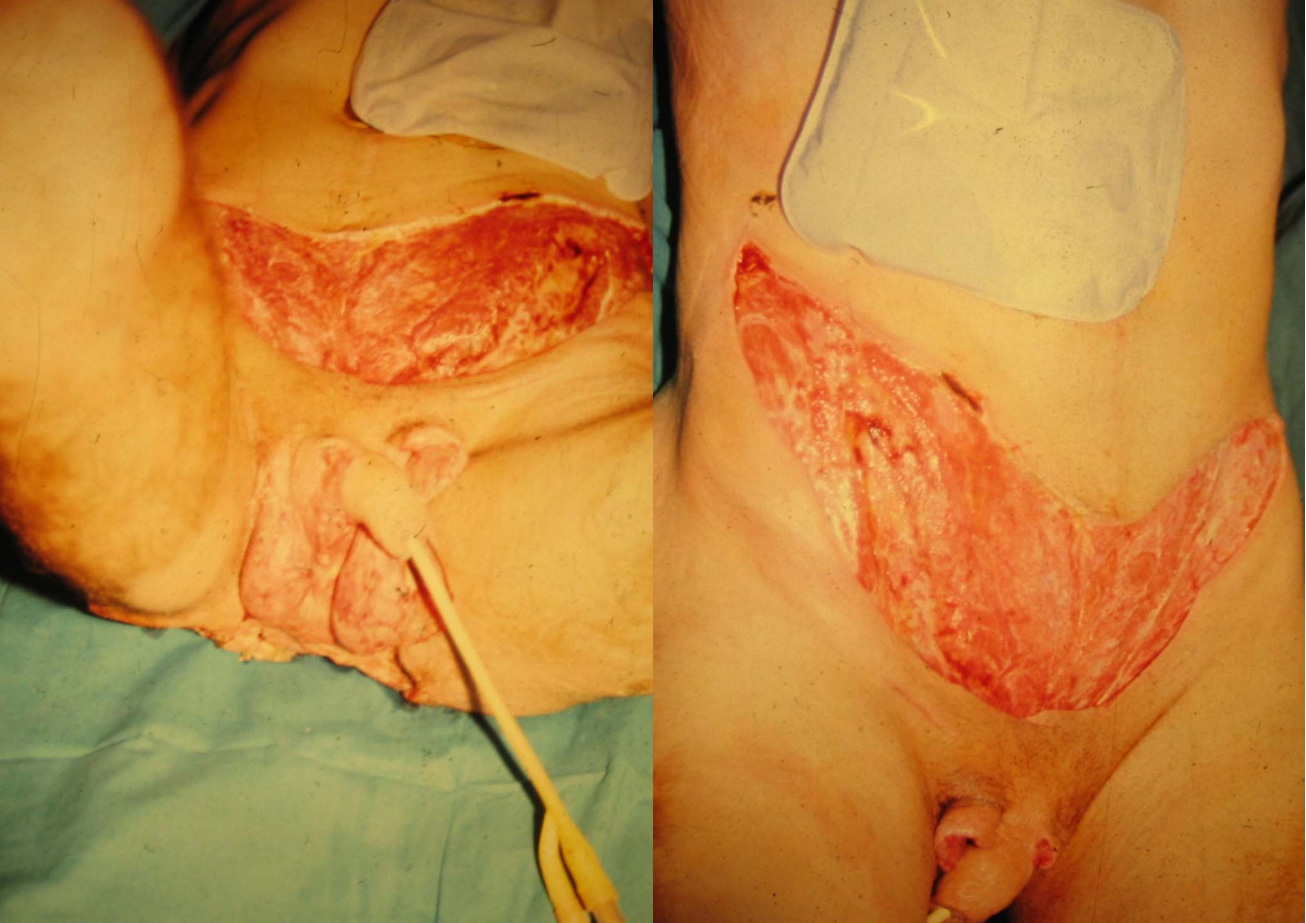
.**A view of the abdominal wall from case III before second stage reconstruction of the soft tissue defects.**

NF after abdominal surgery or spreading infections from the perineum or the lower extremities is extremely serious with great defects and carries a high morbidity and mortality rate (Figure 2). The goals of the reconstructive surgery in the management of complex AW defects (AWD) is to restore the structural and functional continuity of the muscle-fascial system, provide stable coverage and achieve local wound closure [60]. The size of the wound defect after NF of the abdominal wall typically depends on the type of infection and the way it spreads. For reconstructive purposes, AWD can be divided into midline or lateral, and to the upper, middle, or lower third of the abdomen. The most useful method for ventral hernia repair with AWD is the use of *"Component separation technique" *by Ramirez and coworkers [61]. They used muscle-fascial components of the AW in continuity with their vascular and nerve supply to restore ventral defects. Midline partial defects of the skin and deep structures can be repaired in several ways. Firstly, we can use primary closure and skin grafts. The next option is a synthetic mesh [51], which cannot be used on the infected field. It comes in various sizes and shapes at low cost. Biological meshes [52] are resistant to infection, allow natural remodeling, potential stretching, are expensive and are of limited size. Further options include the component separation technique, free, local or distant flaps, TNP therapy, and tissue expansion [60]. A combination of all these techniques is also possible. The reconstruction of the structural components of the AW is an important issue, but even more important is the restoration of the AW function. Midline complete defects can be repaired in similar fashion, because the defects include both skin and fascia, which often require component separation technique, biologic mesh, the local flaps with or without tissue expansion. Lateral defects are more often repaired using direct closures, skin grafts, local advancement flaps, distant flaps, or TNP therapy [60].

## Conclusion

Necrotizing infections refer to rapidly spreading infections, usually located in the fascial planes of soft tissue areas, that result in extensive tissue necrosis, severe sepsis, wide spread organ failure and death. The early diagnosis and management of NF is essential since delayed disease recognition and lack of effective treatment modalities increase the mortality of NF [1].

In everyday surgical practice infections that are life threatening conditions and which require early recognition and aggressive surgical debridement along with broad spectrum antibiotics therapy, are rare. When NF becomes a rapidly progressing necrosis of the subcutaneous fat and fascia, it develops into a life threatening disease that needs prompt recognition, extensive debridement, immediate antibiotic therapy and intensive care treatment. Early and aggressive surgery is mandatory for establishing the right diagnosis as well as for removing as much infected tissue as possible in a single operation. The diagnosis remains primarily clinical, but diagnostic adjuncts such as LRINEC scoring system can be useful for early and precise diagnosis [5].

Different types of microorganisms can cause NF. As seen in our clinical study, the majority of cases begin with an existing infection, most frequently on the extremities, in the perineum or on the AW.

As previously stressed, the treatment modalities of NF in different patient groups are very heterogeneous, but the most important factor of mortality is the time of operative intervention, as well as the number of co-morbidities [36]. Patients with DM appear to be particularly at risk, representing over 70% of cases in one large review [46]. The other co-morbidities include obesity, alcohol abuse, immune-deficiency, chronic renal failure, liver cirrhosis, hypertension, peripheral vascular disease and age above 60 years [1,2].

In cases where the diagnosis is uncertain, repeated clinical assessment and multiple vectors approach integrating a range of diagnostic modalities will optimize the final diagnosis [1]. Many physicians today are not familiar enough with NSTI and NF to proceed rapidly with an accurate diagnosis and the necessary management [36]. The majority of cases today are treated on an outpatient basis or in outpatient clinics. On the other hand, each untreated necrotizing infection or a misdiagnosed case has a poor prognosis and severe course.

In highly suspicious cases of necrotizing infections a multidisciplinary team approach is mandatory, involving the GP doctor, general and plastic surgeons, radiologists, microbiologists, physiotherapists and nutritionists. In the majority of clinical cases, surgeons have a high responsibility level for timely and appropriate surgical treatment and therefore the final outcome. Thus, early surgical debridement, combined with broad spectrum antibiotics, intensive care therapy and adjuvant HBO therapy should become part of the *"Treatment doctrine for NSTI and NF"*, as well as for the treatment of clostridial myonecrosis [36].

### Patient Consent

Written informed consent was obtained from the patients for publication of this case report and accompanying images. A copy of the written consent is available for review by the Editor-in-Chief of this journal.

## Abbreviations

AB: antibiotics; ATA: atmospheres absolute pressure; AW: abdominal wall AWD: abdominal wall defect; CRP: C-reactive protein; CW: chest wall; DM- Diabetes mellitus; GP: General practitioner; HBOT: Hyperbaric oxygen therapy; ICU: Intensive care unit; LRINEC: laboratory risk indictor for necrotizing fasciitis; MODS: multiple organ dysfunction syndrome; MRSA: meticillin resistant *Staphylococcus aureus*; NF: Necrotizing fasciitis; NSTIs: Necrotizing soft tissue infections; PMNs: Polymorphonuclear neutrophils; RS: retroperitineal space; SG: skin graft; SIRS: systemic inflammatory response syndrome; SSTIs: Skin and soft tissue infections; TNP: Topical negative pressure.

## Competing interests

The authors declare that they have no competing interests.

## Authors' contributions

All authors participated in the design of the paper, conceived the paper, and participated in drafting and critical revision for important intellectual content. All authors read and approved the final form of this manuscript.
